# Comparison of Postoperative Analgesia in Patients Undergoing Ileostomy Closure with and Without Dual Transversus Abdominis Plane (TAP) Block: A Randomized Controlled Trial

**DOI:** 10.5041/RMMJ.10356

**Published:** 2019-01-28

**Authors:** Abhijit Nair, Veena Enagandula Amula, Vibhavari Naik, Praveen Kumar Kodisharapu, Anne Poornachand, M. S. Shyam Prasad, Mohammed Salman Saifuddin, Basanth Kumar Rayani

**Affiliations:** Department of Anesthesiology, Basavatarakam Indo-American Cancer Hospital and Research Institute, Hyderabad, Telangana, India

**Keywords:** Anesthesia, colostomy, local anesthesia, nerve block, transversus abdominis, ultrasound

## Abstract

**Background and Aims:**

Multimodal analgesia comprising opioid, paracetamol, and non-steroidal anti-inflammatory drugs is used for managing postoperative surgical pain after ileostomy closure (IC). We investigated the efficacy of unilateral dual transversus abdominis plane (TAP) block to reduce morphine consumption in the first 24 hours along with a reduction in visual analogue score for pain and in postoperative nausea/vomiting.

**Methods:**

This was a single-center, investigator-initiated, prospective, parallel-group, placebo-controlled randomized study involving patients undergoing IC under general anesthesia. We recruited 55 patients in two groups: 28 in a TAP group and 27 in a placebo group. The TAP group patients received 30 mL of 0.375% bupivacaine: 15 mL by a posterior TAP approach and 15 mL by a subcostal approach using ultrasonography. Patients in the placebo group received 30 mL normal saline (placebo) using the same approaches. Blocks were administered at the end of surgery before extubation. To monitor for the primary outcome—24-hour morphine consumption for both groups—patients were transferred to a high-dependency unit. The secondary outcome was to compare postoperative nausea/vomiting in both groups.

**Results:**

The demographic data, gender distribution, ASA physical status, duration of surgery, and time of first morphine dose was comparable in both groups. The 24-hour morphine consumption was 3.29±2.78 mg and 9.23±2.94 mg for the TAP and placebo groups, respectively, which was statistically significant (*P*=0.001).

**Conclusion:**

Dual TAP block reduces opioid consumption in the first 24 hours after an IC and can facilitate early recovery with less adverse effects seen than with opioids and NSAIDs.

## INTRODUCTION

Diversion ileostomy is a surgical procedure performed to protect an anastomotic leak after a colorectal anastomosis. It is selectively used in high-risk patients who have a low pelvic anastomosis that is at increased risk of anastomosis leak.^1^ A diversion ileostomy is usually closed after 6–8 weeks during which the downstream anastomosis is expected to heal.^2^ Ileostomy closure (IC) is usually performed under subarachnoid block or general anesthesia with multimodal analgesia for postoperative pain relief. Surgical patients undergoing subarachnoid block may experience discomfort resulting from peritoneal stretch due to stimulation of Meissner and Auerbach plexuses of the gut wall or intraoperative adhesiolysis.^3^ Multimodal analgesia usually consists of paracetamol, tramadol, and non-steroidal anti-inflammatory drugs (NSAIDs). There are concerns regarding NSAID use in patients undergoing colorectal anastomosis as it can lead to leakage at the anastomotic site.^4^ Opioids are used as mainstay analgesics in such patients, but this can contribute to postoperative constipation and ileus, lengthening hospitalization, and increasing treatment cost. Abdominal wall blocks have been used successfully alone or as a part of multimodal analgesia in patients undergoing supraumbilical, infraumbilical, and major laparotomies.

We hypothesized that adding a dual transversus abdominis plane (TAP) block would reduce morphine consumption and provide good postoperative analgesia with less postoperative nausea and vomiting (PONV) in patients undergoing ileostomy closure.

## METHODS

This was a single-center, investigator-initiated, prospective, parallel-group, placebo-controlled randomized study that received Hospital Ethics Committee approval (no. IEC/2017/29). The study was registered with the Clinical Trials Registry of India (no. CTRI/2017/03/08192).

**Figure 1:**
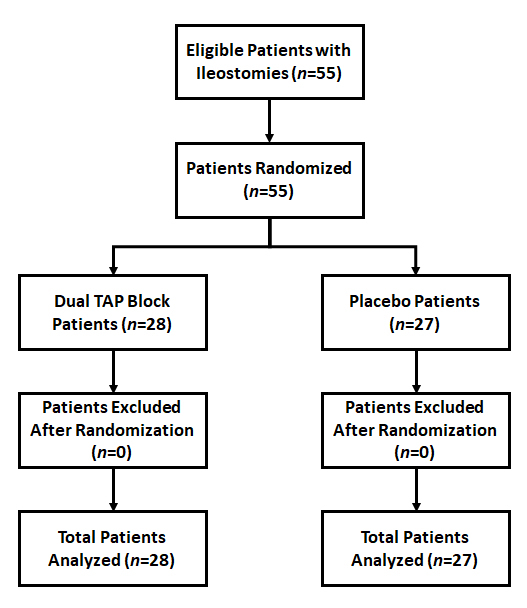
CONSORT Flow Diagram of Patients Enrolled in the Randomized Controlled Trial.

Patients with American Society of Anesthesiologists (ASA) physical status I and II, between 18 and 80 years of age, and undergoing ileostomy closure were included in the study. Patients were included only after signing an informed consent. Exclusion criteria included any patient unable to give informed consent; unwilling to participate; with a history of relevant drug allergy; currently taking analgesics; suffering from current acute or chronic pain or receiving medical therapies for such pain, considered to result in opioid tolerance; and/or patients with an abnormal coagulation profile. The CONSORT flowchart is depicted in Figure 1. The documented consent was provided in the language documented consent was provided in the language best understood by the patient and included a description of the study purpose, protocol, risks, benefits, and an option to exit the study at any point. The participants were assured that, irrespective of the group to which they were assigned, their postoperative pain would be managed effectively.

**Figure 2:**
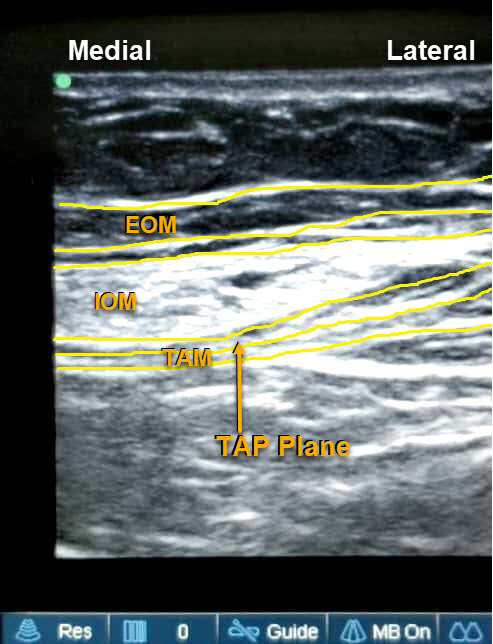
Figure Showing Sonoanatomy of Muscles Relevant for Posterior TAP Block EOM, external oblique muscle; IOM, internal oblique

**Figure 3:**
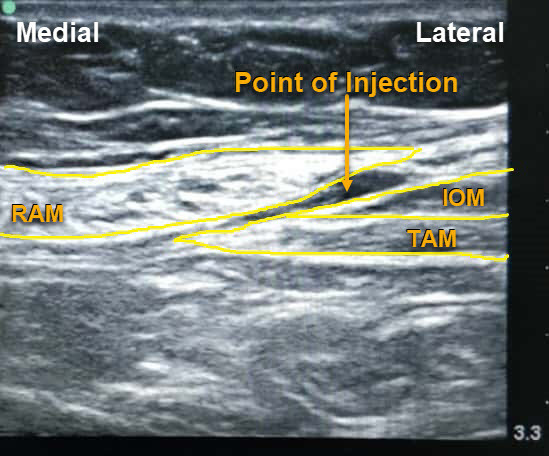
Figure Showing Sonoanatomy of Muscles Relevant for Subcostal TAP Block IOM, internal oblique muscle; RAM, rectus abdominis muscle; TAM, transversus abdominis muscle.

All patients were evaluated for fitness at a pre-anesthesia check-up clinic by the anesthesiologist. Patients fasted for 6 hours prior to surgery. Computer-generated randomization was performed for both groups (www.random.org). Patients were prepped in the operating room according to standard procedure, and intravenous (IV) midazolam (0.03 mg/kg) and fentanyl (1.5 μg/kg, maximum 100 μg/kg) premedication was administered. General anesthesia was induced with IV propofol 2–2.5 mg/kg and the airway secured with an appropriately sized cuffed endotracheal tube, after achieving a neuromuscular block with IV vecuronium bromide (0.1 mg/kg). General anesthesia was maintained with oxygen–medical air and isoflurane using volume-controlled ventilation, and dial concentration was adjusted to target a minimum alveolar concentration of 1.0. The end-tidal carbon dioxide was monitored using a capnograph targeted to 35–40 mmHg. During skin closure, 1 g IV paracetamol was administered. As per randomization, TAP block with drug or placebo was performed by a posterior and subcostal approach. In the TAP group 28 patients received 30 mL 0.375% of bupivacaine by two approaches: 15 mL by subcostal approach and 15 mL by posterior TAP under ultrasound guidance. The 27 patients in the placebo group received 30 mL normal saline by the same approaches. A high-frequency linear array probe (Sonosite M-Turbo Inc., Bothell, WA, USA) was used to perform the block. For posterior TAP block, the probe was placed at the midpoint of the subcostal margin and anterior superior iliac spine along the mid-axillary line. The probe was moved to visualize the external oblique muscle, the internal oblique muscle, and the transversus abdominis muscle (TAM) (Figure 2). With an in-plane approach, a 50 cm insulated needle was inserted from the medial to lateral side until it reached the fascial plane between the internal oblique muscle and TAM. After hydrodissection with 2 mL normal saline to confirm the correct needle 2 mL normal saline to confirm the correct needle position, 15 mL of the study drug, as per randomization, was injected after negative aspiration of blood. The probe was then placed along the subcostal margin to identify the rectus abdominis muscle (RAM), then moved laterally to identify the TAM below RAM (Figure 3). The injection was administered in the fascial plane between these two muscles, with the needle in-plane from the medial to lateral side.

After the block, the patients were extubated after reversing neuromuscular blockade with 0.05 mg/kg neostigmine and 0.01 mg/kg glycopyrrolate. They were then transferred to a high-dependency unit. The visual analogue scale was used to assess pain postoperatively. Intravenous paracetamol 1 g every 6 hours was continued in the postoperative period for patients above 50 kg and 1 g every 8 hours for patients less than 50 kg. Intravenous morphine was used as rescue analgesic in both groups, if the patient’s VAS score was more than 4, as follows: 2 mg IV for patients 50 kg or less, and 3 mg IV for patient over 50 kg. The PONV and total morphine consumption for the 24-hour period was recorded. The primary outcome of this study was to compare total morphine consumption after IC between both groups at the end of 24 hours, with the secondary outcome being a comparison of PONV in both groups.

## RESULTS (TABLE 1)

Statistical Package for the Social Sciences (SPSS) version 21 (IBM Corp., Armonk, NY, USA) was used for statistical analysis. A total of 55 patients were randomized into two groups, with 28 patients in the TAP group and 27 patients in the placebo group. Mean age and mean weight were comparable in both groups, as was gender distribution (male and female patients). There was no statistical difference between ASA physical status, intraoperative fentanyl use, and surgery duration in either group (*P* values 0.218, 0.169, and 0.324, respectively). There was no statistical difference between vital parameters, i.e. heart rate, mean systolic blood pressure, mean diastolic pressure, and mean arterial pressure at baseline and immediate postoperatively after extubation. The time of first morphine dose after surgery and the PONV score were compared using the Mann–Whitney *U* test in both groups. There was no statistical difference between the first morphine dose after surgery and the PONV score between both groups (*P* value 0.130 and 0.061, respectively). Total morphine consumption in 24 hours was analyzed using the Mann–Whitney *U* test. Mean (±SD) 24-hour morphine consumption in the TAP group was 3.29±2.78 mg and 9.23±2.94 mg for the placebo group, which was statistically significant (Table 1; *P*=0.001).

**Table 1:** Table Comparing Demography, Duration of Surgery, Intraoperative Fentanyl Used, and First Morphine Dose, 24-h Morphine Consumption, PONV Score, and Baseline Parameters in Both Groups.

Variable	Group (Mean±SD)		*P* Value
	Transversus Abdominis Plane (*n*=28)	Placebo (*n*=27)	
Sex, M/F	10/18	17/10	0.043†
Age	51.82±12.16	49.33±11.51	0.440*
Weight (kg)	54.32±13.62	60.89±12.96	0.073*
ASA (I/II)	13/15	17/10	0.218†
Duration of Surgery (min)	146.79±23.57	141.11±18.25	0.324*
Intraoperative Fentanyl Used (μg)	152.68±28.33	163.89±31.26	0.169*
First Morphine Dose (min)	290.36±450.48	138.89±248.38	0.130‡
PONV Score	0.29±1.049	1.04±1.786	0.061‡
24-h Morphine Consumption (mg)	3.29±2.78	9.23±2.94	0.001‡
Baseline Parameters			
Baseline HR	82.79±11.77	81.81±8.709	0.730*
Immediate Postoperative HR	79.86±11.85	78.44±13.63	0.683*
Baseline SBP	132.32±17.19	128.96±16.15	0.459*
Immediate Postoperative SBP	134.75±13.16	139.37±18.43	0.288*
Baseline DBP	80.46±8.54	75.81±8.94	0.054*
Immediate Postoperative DBP	81.86±7.64	78.19±11.15	0.159*
Baseline MAP	97.54±10.65	93.22±10.20	0.131*
Immediate Postoperative MAP	98.43±9.46	98.26±12.002	0.954*

* Unpaired *t* test.

† Chi-square test.

‡ Mann–Whitney *U* test.

ASA, American Society of Anesthesiologists physical status; DBP, diastolic blood pressure; HR, heart rate; MAP, mean arterial pressure; PONV, postoperative nausea and vomiting; SBP, systolic blood pressure.

## DISCUSSION

The nerve supply to the skin, muscles, and parietal peritoneum of the anterior abdominal wall is from the anterior rami of the T6–T12 thoracic nerves and the ﬁrst lumbar nerve (L1). These nerves exit their respective intervertebral foramina, cross the vertebral transverse process, and pierce the musculature of the anterior and lateral abdominal wall. Branches from the anterior rami of T6–L1 include the intercostal nerves, the subcostal nerve, and the ilioinguinal and iliohypogastric nerves. Each segmental nerve has a sensory branch at the mid-axillary line. These thoracolumbar nerves traverse through the lateral abdominal wall in the fascial plane between the internal oblique muscle and the TAM. This myofascial plane is referred to as the TAP. The sensory nerves have a lateral cutaneous branch at the mid-axillary line and then continue into the TAP, supplying the abdominal wall until the midline.^5^

Using abdominal blocks for pain relief began in 1993 when Kuppuvelumani et al. performed bilateral abdominal blocks to provide pain relief from T10–L1 in 30 patients who underwent caesarean section.^6^ The authors administered two injections: the first was 1 cm medial to the anterior superior iliac spine and the second just below the tenth rib along the anterior axillary line. In 2001, Rafi described the TAP block as a peripheral nerve block administered in the plane between the internal oblique muscle and TAM.^7^ It was described as a landmark-guided technique, and injection was performed after a characteristic double pop. The landmarks were quite inconsistent and were not particularly reliable after surgery. However, with the increased use of ultrasound in regional anesthesia techniques, TAP block became popular and is considered a standard of care by many practicing anesthesiologists in the multimodal approach to manage surgical pain after abdominal surgeries. The TAP block is not superior to epidural analgesia or intrathecal morphine, but when administered in the correct plane with a good volume and concentration of local anesthetic it is morphine-sparing in almost all lower abdominal surgeries.^8^

In 2007, McDonnell et al. used TAP block in patients undergoing large-bowel resection and found it to be effective in managing acute postoperative pain with reduced morphine consumption.^9^ Sedation and PONV due to opioids were reduced when TAP block was used for abdominal surgeries.^10^ In 2008, Hebbard et al. described a subcostal TAP block in which they injected local anesthetic into the TAP lateral to the linea semilunaris immediately inferior and parallel to the costal margin.^11^ The authors found the block to be effective for surgeries in the supraumbilical area (T6–T9), the area spared by posterior TAP block. Ma et al. defined the areas covered by subcostal a TAP block as the anterior abdominal wall between the medioventral line to the anterior axillary line excluding the lateral upper abdominal region.^12^

Different approaches to TAP block have been described, namely lateral, posterior, subcostal, and oblique subcostal. The dual TAP block that we used for IC was described earlier by Børglum et al. for major open or laparoscopic abdominal surgeries.^13^ The authors referred to the dual block as a medial intercostal TAP block. Although the dual block is known to cover somatic pain and has less efficacy against visceral pain, in our study we observed good pain relief for patients in the TAP group. Tsai et al. classified TAP block based on the involved spinal nerves instead of the probe positions,^14^ i.e. the subcostal, lateral/posterior, and dual approaches. A subcostal TAP provides analgesia to the upper abdomen (T6–9); a lateral or a posterior TAP block provides analgesia to the lower abdomen (T9–12); and a dual block, which is the combined subcostal and lateral/posterior TAP block, covers the entire upper and lower abdomen on one side. The TAP block has evolved over the years from a landmark-guided technique to a peripheral nerve block with many approaches and nomenclature.^15^

Several studies have shown that TAP block addresses somatic pain only, and thereby has a limited role when visceral pain is the issue. Wu et al. felt that although a single-shot TAP block was better than IV opioid analgesia, continuous thoracic epidural analgesia was more effective than a single-injection subcostal TAP block.^16^ However, TAP block is quite simple to learn and master, has fewer complications, and has good results when using ultrasound guidance.^17^ The TAP block has significantly long-lasting analgesia effects compared to local anesthetic infiltration at the surgical site.^18^ The efficacy of the TAP block is comparable to IV morphine patient-controlled analgesia when used for lower abdominal surgeries but with fewer ileus and sedation events.^19^ Its analgesic efficacy depends on the approach used for injecting local anesthetic. Contrast studies have shown that there was no spread to the paravertebral space with the anterior subcostal approach, whereas with a mid-axillary TAP block there is little contrast enhancement in the paravertebral space at T12–L2. The posterior approach leads to contrast spread around the quadratus lumborum to the paravertebral space from the T5–L1 vertebral levels.^20^–^22^

**Figure 4:**
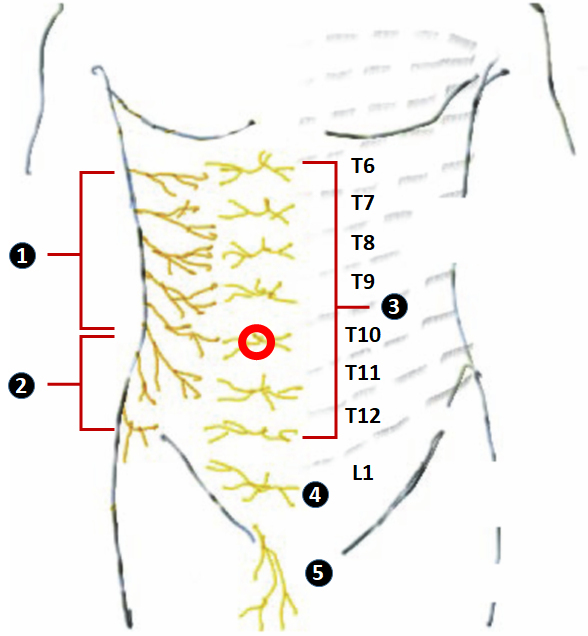
Figure Showing Dermatomes Involved during Ileostomy Closure ❶ Lateral cutaneous branches of T6-11; ❷ lateral cutaneous branches of T12; ❸ Anterior cutaneous branches of thoracic nerves (T6-12); ❹ Anterior cutaneous branches of iliohypogastric nerve (L1); ❺ ilioinguinal nerve (L1); and red circle ( 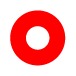), location of diversion ileostomy. Figure modified from Tsai et al.,^14^ with permission [Creative Commons Attribution License].

The meta-analysis by Charlton et al. in the Cochrane Database Systematic Review (2010) concluded that there is limited evidence to indicate that use of perioperative TAP block reduces opioid consumption and pain scores after abdominal surgery when compared to no intervention or placebo, with no significant PONV or sedation reduction.^23^ However, since 2010, i.e. after that meta-analysis was published, several randomized trials have been published proving their conclusion was incorrect. This was highlighted by the systematic review and meta-analysis published by Brogi et al. in 2016, which concluded that TAP block can play an important role in pain management after abdominal surgery by reducing both pain scores and 24-hour morphine consumption, especially when neuraxial techniques or opioids are contraindicated.^24^ The innervation involved for an IC is from dermatomes T6–T10, and a single-shot posterior TAP cannot cover this area (Figure 4). We therefore opted for a dual-block, i.e. a subcostal TAP covering T6–T9 and a posterior TAP that covers the T9–T12 dermatomes. For this reason, we hypothesized that a dual TAP block would provide comprehensive unilateral analgesia after IC. Abreu et al. found that problems like postdural puncture headache, urinary retention, and ileus were more often encountered in patients undergoing IC with subarachnoid block.^25^

There are a few limitations to our study. The block was performed after the surgery. In some patients, particularly thin ones, we noted that the myofascial plane was obscured due to surgical dissections, making it difficult to identify the muscles. This could be avoided by performing the block immediately after anesthesia induction. This would also reduce the intraoperative anesthetic and opioid requirement. Secondly, we did not follow up either group of patients to determine the time of first flatus and defecation.

## CONCLUSION

In conclusion, a dual TAP block is a reliable addition to the armamentarium of multimodal analgesia for patients undergoing IC. Good pain relief can facilitate early mobilization, reduced PONV, early recovery of gut function, and early discharge from the hospital. Use of NSAIDs and opioids can also be avoided in managing postoperative pain of these patients.

## Abbreviations

ASA: American Society of Anesthesiologists
IC: ileostomy closure
IV: intravenous
NSAIDs: non-steroidal anti-inflammatory drugs
PONV: postoperative nausea and vomiting
RAM: rectus abdominis muscle
SD: standard deviation
TAM: transversus abdominus muscle
TAP: transversus abdominis plane.
